# Assessing the Quality and Reliability of ChatGPT’s Responses to Radiotherapy-Related Patient Queries: Comparative Study With GPT-3.5 and GPT-4

**DOI:** 10.2196/63677

**Published:** 2025-04-16

**Authors:** Ana Grilo, Catarina Marques, Maria Corte-Real, Elisabete Carolino, Marco Caetano

**Affiliations:** 1 Research Center for Psychological Science of the Faculty of Psychology, University of Lisbon to CICPSI, Faculdade de Psicologia, Universidade de Lisboa, Av. D. João II, Lote 4.69.01, Parque das Nações, Lisboa, 1990-096, Portugal, 351 964371101; 2 Escola Superior de Tecnologia da Saúde, Instituto Politécnico de Lisboa, Lisboa, Portugal

**Keywords:** artificial intelligence, ChatGPT, large language model, radiotherapy, patient information, quality, internet access, health information, cancer awareness, accuracy, readability, chatbot, patient query, chat generative pretrained transformer, OpenAI, natural language processing, patients with cancer

## Abstract

**Background:**

Patients frequently resort to the internet to access information about cancer. However, these websites often lack content accuracy and readability. Recently, ChatGPT, an artificial intelligence–powered chatbot, has signified a potential paradigm shift in how patients with cancer can access vast amounts of medical information, including insights into radiotherapy. However, the quality of the information provided by ChatGPT remains unclear. This is particularly significant given the general public’s limited knowledge of this treatment and concerns about its possible side effects. Furthermore, evaluating the quality of responses is crucial, as misinformation can foster a false sense of knowledge and security, lead to noncompliance, and result in delays in receiving appropriate treatment.

**Objective:**

This study aims to evaluate the quality and reliability of ChatGPT’s responses to common patient queries about radiotherapy, comparing the performance of ChatGPT’s two versions: GPT-3.5 and GPT-4.

**Methods:**

We selected 40 commonly asked radiotherapy questions and entered the queries in both versions of ChatGPT. Response quality and reliability were evaluated by 16 radiotherapy experts using the General Quality Score (GQS), a 5-point Likert scale, with the median GQS determined based on the experts’ ratings. Consistency and similarity of responses were assessed using the cosine similarity score, which ranges from 0 (complete dissimilarity) to 1 (complete similarity). Readability was analyzed using the Flesch Reading Ease Score, ranging from 0 to 100, and the Flesch-Kincaid Grade Level, reflecting the average number of years of education required for comprehension. Statistical analyses were performed using the Mann-Whitney test and effect size, with results deemed significant at a 5% level (*P*=.05). To assess agreement between experts, Krippendorff α and Fleiss κ were used.

**Results:**

GPT-4 demonstrated superior performance, with a higher GQS and a lower number of scores of 1 and 2, compared to GPT-3.5. The Mann-Whitney test revealed statistically significant differences in some questions, with GPT-4 generally receiving higher ratings. The median (IQR) cosine similarity score indicated substantial similarity (0.81, IQR 0.05) and consistency in the responses of both versions (GPT-3.5: 0.85, IQR 0.04; GPT-4: 0.83, IQR 0.04). Readability scores for both versions were considered college level, with GPT-4 scoring slightly better in the Flesch Reading Ease Score (34.61) and Flesch-Kincaid Grade Level (12.32) compared to GPT-3.5 (32.98 and 13.32, respectively). Responses by both versions were deemed challenging for the general public.

**Conclusions:**

Both GPT-3.5 and GPT-4 demonstrated having the capability to address radiotherapy concepts, with GPT-4 showing superior performance. However, both models present readability challenges for the general population. Although ChatGPT demonstrates potential as a valuable resource for addressing common patient queries related to radiotherapy, it is imperative to acknowledge its limitations, including the risks of misinformation and readability issues. In addition, its implementation should be supported by strategies to enhance accessibility and readability.

## Introduction

In an increasingly digitized society, patients frequently resort to the internet to access information about cancer [1-3]. However, despite being one of the most favored informational modalities, websites often require more content accuracy and better readability [1].

Recently, artificial intelligence (AI)–powered chatbots such as ChatGPT have signified a potential paradigm shift in how patients with cancer can access a vast amount of medical information [1,3,4]. The rise of these AI platforms, accessible to the general public, has escalated notably since OpenAI released version 3.5 of ChatGPT (GPT-3.5) on November 30, 2022 [4-13], which amassed over 1 billion users in March 2023 [4].

ChatGPT, a large language model (LLM) [6,14-17], uses natural language processing to offer varied responses to the same query considering the context of the conversation and individual user preferences [18]. Through text-to-text communication, ChatGPT can engage with humans [12] and aims to deliver responses resembling human interactions [6,14,18]. This model has undergone extensive training on a diverse corpus of text data encompassing a broad spectrum of sources, including books, scholarly articles, and web pages, enabling it to effectively comprehend and respond to natural language queries across a broad range of topics [19,20]. Moreover, the model’s performance is enhanced through reinforcement learning from human feedback, which enables it to produce more coherent and contextually relevant responses [21]. Additionally, ChatGPT can compose emails, essays, and medical reports, as well as solve problems and provide clarification [10,13,22,23].

On March 14, 2023, OpenAI announced the release ChatGPT-4 (GPT-4), which became available through a subscription-based model [9,12,16]. This new version demonstrated outstanding performance across numerous academic and professional benchmarks, providing more refined and varied responses than GPT-3.5 [24].

In this context, ChatGPT has emerged as a contender for traditional search engines, such as Google, because of its capacity to filter vast quantities of data and provide easily comprehensible responses [4,6]. Consequently, ChatGPT is a potentially reliable source of medical information to both the public and patients with cancer, and it is capable of offering insights regarding radiotherapy [4,25]. This is particularly significant given the general public’s limited knowledge of this treatment [15,26] and concerns regarding its possible side effects [27].

Radiotherapy is a well-established treatment that delivers targeted ionizing radiation with precision with the aim of destroying cancer cells while minimizing damage to healthy tissues. Approximately half of all patients diagnosed with cancer undergo radiotherapy as a part of their care. Advances in radiotherapy have increased its complexity, requiring greater preparation and support for patients who may face physical and psychological challenges [28]. Considering that approximately 80% of patients have limited knowledge regarding radiotherapy and associated expectations regarding treatment, many have significant misconceptions. These commonly include concerns about radiation burns or the possibility of becoming radioactive as a result of the treatment [29,30]. Such misunderstandings, coupled with the unfamiliarity of radiotherapy for most patients and the inherent invisibility of the treatment, further complicate their ability to fully comprehend the process [31,32]. Therefore, providing clear and accessible information is essential for reducing patients’ fear of treatment [31]. Previous studies have explored radiotherapy educational resources, such as videos, and tested group education in radiotherapy settings. However, these studies did not specifically address individual patient education and support needs at key time points [33]. Alternatively, written documentation has proven effective for patients who may feel overwhelmed by excessive verbal information, as it allows them to process the material at their own pace and share it with family and friends [34]. Therefore, ChatGPT offers a convenient and accessible method for patients to obtain written information and support [19].

Given that patient education is particularly crucial for patients with cancer because of the complexity of their treatment pathways [20], providing them with comprehensive information about radiotherapy at appropriate stages may enhance adherence to the treatment plan, because inadequate information can lead to increased uncertainty, unnecessary anxiety, and distress among patients and their families [27,35,36]. Additionally, poorly informed patients are likely to be dissatisfied with their care, have difficulty coping [35], and have many follow-up questions regarding the treatment process. Moreover, patients with cancer often feel uncomfortable discussing their body image and sexual health with their clinicians. Consequently, patient communication with ChatGPT may lower these barriers [36].

However, given that ChatGPT was not explicitly trained for oncology-related inquiries, the quality of the information it provides remains unverified [7,14,36]. Evaluating the quality of responses is crucial, as misinformation can foster a false sense of knowledge and security, lead to noncompliance, and result in delays in receiving appropriate treatment [4,14,15]. Nevertheless, various limitations of ChatGPT have been identified. It has been observed to fall below the expected educational level [4], as health-related materials intended for patient consumption are typically recommended to have a reading level equivalent to fifth and sixth grades [4,37]. Furthermore, the training data for GPT-3.5 are outdated, limited to the information available up until September 2021, and lack access to newer knowledge beyond that date [5,38,39]. To address this constraint, GPT-4 introduces a novel feature that allows the use of external plug-ins [25]. However, this new version is available exclusively through paid subscription [9,12,16]. Additionally, ChatGPT tends to provide unreliable or inaccurate information, potentially generating incorrect or misleading responses [14,16]. This issue often arises from the dependence of models on their training data, which may not always be up-to-date or fully comprehensive [40].

To date, limited research has been conducted on the application of language models in the medical domain and the effectiveness of ChatGPT in patient education remains indeterminate [14]. Although the literature addressing ChatGPT’s capabilities has proliferated in recent months, there remains a lack of data regarding the quality and reliability of the responses it provides [11,18]. This gap underscores the necessity for more comprehensive studies to evaluate the performance of language models, including ChatGPT, in the medical context. Ensuring that these models are equipped with the most current and comprehensive data is essential for their effective application in radiotherapy health care.

This study aimed to evaluate the quality and reliability of ChatGPT responses to common patient queries regarding radiotherapy to ascertain its potential as a reliable source of patient information. Additionally, it aimed to compare the performance of GPT-3.5 with GPT-4 in generating responses to the same radiotherapy queries.

## Methods

### Prompt Generation

To determine the most common patient queries regarding radiotherapy, an assessment was conducted using articles that addressed topics related to the most relevant patient concerns. These served as the foundation for the development of 128 questions, 90 of which were derived from the studies by Halkett et al [27,35,41] and Zeguers et al [32], whereas the remaining 38 were sourced from the National Cancer Institute [42]. The questions were then organized into a table to facilitate the identification of duplicates and the selection of the most pertinent ones. There were 36 questions identified as duplicates, and 43 were deemed specific to certain pathologies or specialized treatments, leaving a total of 49 questions. Four authors (AG, CM, MC-R, and MC) excluded 9 additional questions upon agreement, resulting in a final set of 40 queries to be input into ChatGPT. This exclusion aimed to ensure that the responses could be applied to all patients receiving radiotherapy, thereby reflecting their primary concerns and doubts. The questions were intentionally phrased in the first person to mirror the way patients might typically frame their queries when interacting with ChatGPT [43] and were structured to address the informational needs of patients at various stages of radiotherapy [27]. The final set of questions was categorized into three dimensions: general information (n=14), planning and treatment (n=16), and side effects (n=10) (Textbox 1). These dimensions were selected based on previous studies [27,31,32,44], which assessed the most critical information needs for patients receiving radiotherapy, and they were further chosen to evaluate the strengths and limitations of responses across various topics in radiotherapy.

Textbox 1. **Common patient queries regarding radiotherapy by dimension inserted in ChatGPT.**

**General information**

1. Why is radiotherapy recommended?

2. What does radiotherapy involve?

3. When should radiotherapy and chemotherapy be combined?

4. What’s the cost of radiotherapy treatment?

5. Who will be providing my radiotherapy treatment?

6. How does the radiotherapy treatment machine work?

7. What impact will radiotherapy treatment have on my life?

8. What impact will radiotherapy treatment have on my health in the future?

9. During the period of radiotherapy, will I have to follow a particular diet?

10. Will radiotherapy make me radioactive?

11. What does radiotherapy do to healthy cells?

12. How long does radiotherapy take to work?

13. Can I be cured of my disease through radiotherapy treatments?

14. What will happen after the radiotherapy treatment is finished?

**Planning and treatment**

1. Can I maintain my daily routine and activities during radiotherapy?

2. Can I keep working while undergoing radiotherapy treatments?

3. Are complementary medicines recommended while undergoing radiotherapy treatments?

4. What’s the planning appointment in radiotherapy and what does it involve?

5. Why is computed tomography (CT) planning necessary in radiotherapy?

6. Why are tattoos useful in radiotherapy CT planning?

7. What happens on the first day of radiotherapy treatment?

8. Will the radiotherapy treatment schedule be adjusted to my availability?

9. What am I expected to do during the radiotherapy treatment?

10. Does the radiotherapy machine make noise?

11. How close is the radiotherapy treatment machine going to get?

12. What happens during radiotherapy treatment?

13. Is there a possibility of experiencing pain due to the radiotherapy treatment?

14. How long does a radiotherapy session last?

15. What should I wear for radiotherapy treatment?

16. Will there be follow-up after the end of radiotherapy treatments?

**Side effects**

1. What are the side effects of radiotherapy?

2. What skin care should I have during and after radiotherapy?

3. Am I going to feel tired after the radiotherapy treatments?

4. What hygiene care should be taken after radiotherapy treatments?

5. Which steps should be taken to reduce radiotherapy side effects?

6. Will the radiotherapy treatment be interrupted if I experience adverse side effects?

7. Who can I go to if the radiotherapy side effects become too burdensome?

8. Will radiotherapy affect my fertility?

9. Will radiotherapy cause hair loss?

10. Will radiotherapy cause permanent damage?

### Data Collection

Responses were collected from ChatGPT between April 6, 2024 and April 9, 2024. Each question was queried on both versions of ChatGPT in English. Each query was entered separately using the “New Chat” function, acknowledging that ChatGPT considers the context of the conversation, which can influence responses. Therefore, the memory retention option was disabled when the questions were introduced into ChatGPT to ensure independence of the responses. The queries were then regenerated in each version of ChatGPT, and both responses were documented to analyze consistency.

Various methods were then used, as described in later sections, to assess the quality and reliability of the response content, response consistency, response readability, and similarity between responses from GPT-3.5 and GPT-4.

### Outcomes

#### Quality and Reliability

To evaluate the quality and reliability of the information provided by ChatGPT, we used a 5-point Likert scale, known as the General Quality Score (GQS), which has been used in previous studies [14,45]. The assessment criteria included accuracy, lay-language use, information flow, usefulness, and empathy. The 5-point Likert scale was defined as follows: (1) inaccurate information, poorly organized text, missing important details, and not helpful for patients; (2) limited accuracy, some relevant information is present, but still not easily understandable for patients; (3) adequately accurate information and some important details are explained in plain language; (4) accurate information, well-organized text, and most relevant details are presented in a patient-friendly manner; and (5) extremely accurate information, well-structured text, and all relevant details are presented in a compassionate and patient-friendly manner [14].

The median GQS was calculated by averaging the ratings provided by 16 independent radiotherapy experts with substantial experience in managing oncology patients undergoing radiotherapy. The experts were randomly assigned to evaluate either GPT-3.5 or GPT-4, assuring that each expert evaluated only the responses from one of ChatGPT’s versions to reduce potential bias during the evaluation process, thereby decreasing the likelihood of altering assessments and enhancing their credibility [46]. All the experts received detailed instructions on the evaluation guidelines to promote a uniform understanding of the assessment process. Furthermore, the responses from ChatGPT were provided to the experts in paper format, and their evaluation was conducted in real time without internet access and without knowledge of which version the responses corresponded to, thereby ensuring a blinding effect. Moreover, the authors (CM and MC-R) who analyzed the obtained results were unaware of the identity of the radiotherapy experts.

#### Consistency and Similarity

The consistency and similarity of the responses were evaluated using the cosine similarity score. This method involves transforming the text information provided by ChatGPT into vectors, then calculating the cosine of the angle between the two vectors, indicating how similar the responses are to each other. Scores were calculated using an online tool. The cosine similarity score ranges from 0 to 1, where a score of 0 indicates complete dissimilarity between the texts, and a score of 1 indicates complete similarity [14,36].

To assess the similarity between the responses generated by GPT-3.5 and GPT-4, the initial responses to the same question provided by both versions were inserted into the web-based tool to determine the cosine similarity score between them.

The consistency of the responses generated by ChatGPT was assessed by entering the same question into both versions and calculating the cosine similarity score between the two responses to the same question. By regenerating the same question, we aim to assess whether ChatGPT can provide consistent information or if its responses vary widely.

#### Readability

To evaluate readability, responses from both versions were assessed using a web-based Flesch Reading Ease Score (FRES) calculator. This calculator determined the responses’ readability using two different indices: the FRES and the Flesch-Kincaid Grade Level (FKGL). These readability tests use mathematical formulas that consider factors such as sentence length and word count. The FRES is a numerical score ranging from 0 to 100, with higher numbers indicating better readability, meaning the content is easier to read and understand [8,47,48] and corresponds to a lower grade level [4,47,48] (Multimedia Appendix 1). The FKGL score indicates the average number of years of education needed to comprehend a text, with lower scores suggesting better readability [8,47,48] and correlating to the equivalent school level [4,47] (Multimedia Appendix 2).

### Statistical Analysis

The data were analyzed using SPSS statistical software (version 29.0; IBM Corp). The results were considered statistically significant at the 5% level (*P*=.05). Exploratory data analysis was carried out using frequency analysis (n, %) for categorical variables and median and interquartile range (IQR = Q_3_− Q_1_) for continuous variables. To test the normality of the data, the Shapiro-Wilk test was used. The Mann-Whitney test (since the normality assumption was not verified) and effect size were used to compare the evaluations between the 2 versions of ChatGPT. To analyze the question evaluations, scores were calculated for each question, considering the 8 experts assigned to each version. Krippendorff α and Fleiss κ were used to assess the agreement between experts. For this analysis, the experts’ assessments were considered for all questions in each dimension in each version of ChatGPT.

### Ethical Considerations

This study did not qualify as human subjects research due to the lack of patient involvement and identifying data for the health professionals involved; therefore, it was deemed exempt from institutional review board approval. Additionally, the use of ChatGPT, a public platform accessible to all, meant no permission was required to use the information generated in this study.

## Results

### Quality and Reliability

GPT-3.5 received primarily midrange scores, with most evaluations at levels 3 (n=72) and 4 (n=90), indicating generally accurate and comprehensible responses. Notably, many responses had the highest rating of 5 (n=110), providing extremely accurate and well-structured information. However, they also received low scores of 1 (n=13) and 2 (n=35), suggesting inaccurate or limited information, respectively.

**Figure 1.**
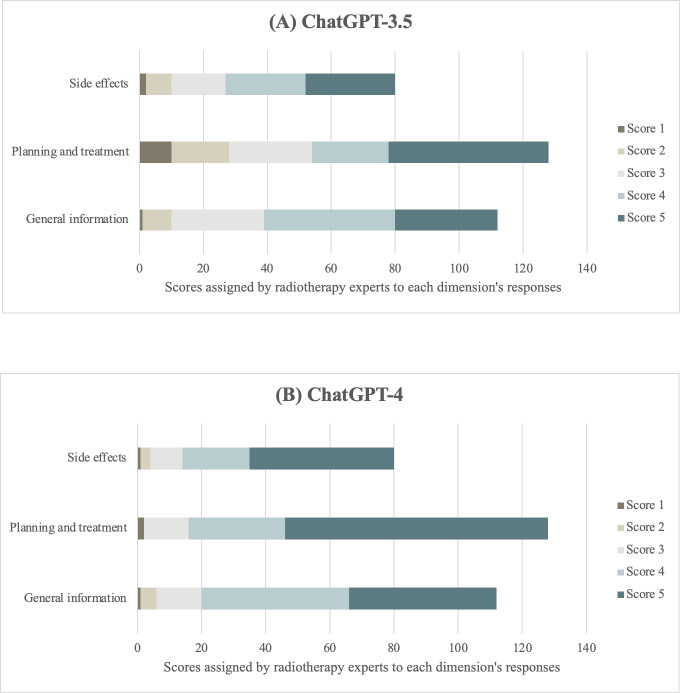
Number of scores assigned by radiotherapy experts to the total number of responses in each dimension from (**A**) ChatGPT-3.5 and (**B**) ChatGPT-4. The Likert scale was defined as follows: score 1=inaccurate information; score 2=limited accuracy; score 3=adequately accurate information; score 4=accurate information; and score 5=extremely accurate information.

Conversely, GPT-4 received the highest score of 5 (n=173), indicating a superior ability to provide accurate and well-structured information. A significant number of responses were assigned a score of 4 (n=97), while a smaller proportion received a score of 3 (n=38), demonstrating that it consistently provided responses that were accurate, well-organized, and accessible to patients. Remarkably, GPT-4 exhibited a lower number of low scores of 1 (n=4) and 2 (n=8*)* than GPT-3.5. The score breakdown by the question dimension is shown in Figure 1.

Considering the general information dimension, statistically significant differences were detected between the 2 versions of ChatGPT regarding questions 3 (*P*=.03, effect size=0.6) and 10 (*P*=.04, effect size=0.5). Regarding planning and treatment, statistically significant differences were detected for questions 5 (*P*=.046, effect size=0.5), 7 (*P*=.002, effect size=0.8), 9 (*P*=.003, effect size=0.7), and 11 (*P*=.02, effect size=0.6). Finally, regarding side effects, statistically significant differences were detected for question 9 (*P*=.04, effect size=0.5). In either situation, GPT-4 showed higher ratings (Table 1). The high effect size values revealed a weak overlap in the response distributions between the 2 versions of ChatGPT. However, in the results of the comparison of the evaluation of the 2 versions of ChatGPT, for the other questions, the effect size was low, revealing overlapping distributions of responses, which is why no statistically significant differences were detected. It can also be seen that, although not significant, version 4 of ChatGPT presents higher evaluation scores.

**Table 1.** Comparison of responses to questions about general information; planning and treatment; and side effects between the 2 versions of ChatGPT, with Mann-Whitney test and effect size results.

Dimension and questions		Number	Mean rank		*P* value	Effect size
			GPT-3.5	GPT-4		
**General information**						
	Q1: Why is radiotherapy recommended?	8	7.56	9.44	.40	0.2
	Q2: What does radiotherapy involve?	8	7.13	9.88	.23	0.3
	Q3: When should radiotherapy and chemotherapy be combined?	8	6.00	11.00	.03	0.6
	Q4: What’s the cost of radiotherapy treatment?	8	9.19	7.81	.53	0.2
	Q5: Who will be providing my radiotherapy treatment?	8	8.13	8.88	.72	0.1
	Q6: How does the radiotherapy treatment machine work?	8	8.25	8.75	.82	0.1
	Q7: What impact will radiotherapy treatment have on my life?	8	7.13	9.88	.20	0.3
	Q8: What impact will radiotherapy treatment have on my health in the future?	8	7.13	9.88	.20	0.3
	Q9: During the period of radiotherapy, will I have to follow a particular diet?	8	7.75	9.25	.44	0.2
	Q10: Will radiotherapy make me radioactive?	8	6.25	10.75	.04	0.5
	Q11: What does radiotherapy do to healthy cells?	8	8.75	8.25	.82	0.1
	Q12: How long does radiotherapy take to work?	8	7.13	9.88	.21	0.3
	Q13: Can I be cured of my disease through radiotherapy treatments?	8	9.69	7.31	.30	0.3
	Q14: What will happen after the radiotherapy treatment is finished?	8	7.25	9.75	.22	0.3
**Planning and treatment**						
	Q1: Can I maintain my daily routine and activities during radiotherapy?	8	7.44	9.56	.24	0.3
	Q2: Can I keep working while undergoing radiotherapy treatments?	8	7.25	9.75	.22	0.3
	Q3: Are complementary medicines recommended while undergoing radiotherapy treatments?	8	9.50	7.50	.26	0.3
	Q4: What’s the planning appointment in radiotherapy and what does it involve?	8	7.00	10.00	.18	0.3
	Q5: Why is computed tomography (CT) planning necessary in radiotherapy?	8	6.25	10.75	.046	0.5
	Q6: Why are tattoos useful in radiotherapy CT planning?	8	7.13	9.88	.23	0.3
	Q7: What happens on the first day of radiotherapy treatment?	8	5.13	11.88	.002	0.8
	Q8: Will the radiotherapy treatment schedule be adjusted to my availability?	8	7.88	9.13	.44	0.2
	Q9: What am I expected to do during the radiotherapy treatment?	8	5.19	11.81	.003	0.7
	Q10: Does the radiotherapy machine make noise?	8	8.00	9.00	.54	0.2
	Q11: How close is the radiotherapy treatment machine going to get?	8	5.75	11.25	.02	0.6
	Q12: What happens during radiotherapy treatment?	8	6.88	10.13	.15	0.4
	Q13: Is there a possibility of experiencing pain due to the radiotherapy treatment?	8	7.56	9.44	.41	0.2
	Q14: How long does a radiotherapy session last?	8	7.44	9.56	.34	0.2
	Q15: What should I wear for radiotherapy treatment?	8	7.00	10.00	.06	0.5
	Q16: Will there be follow-up after the end of radiotherapy treatments?	8	7.38	9.63	.27	0.3
**Side effects**						
	Q1: What are the side effects of radiotherapy?	8	6.75	10.25	.12	0.4
	Q2: What skin care should I have during and after radiotherapy?	8	9.31	7.69	.48	0.2
	Q3: Am I going to feel tired after the radiotherapy treatments?	8	7.50	9.50	.26	0.3
	Q4: What hygiene care should be taken after radiotherapy treatments?	8	6.75	10.25	.10	0.4
	Q5: Which steps should be taken to reduce radiotherapy side effects?	8	7.94	9.06	.63	0.1
	Q6: Will the radiotherapy treatment be interrupted if I experience adverse side effects?	8	8.38	8.63	.91	0.0
	Q7: Who can I go to if the radiotherapy side effects become too burdensome?	8	6.81	10.19	.14	0.4
	Q8: Will radiotherapy affect my fertility?	8	7.63	9.38	.41	0.2
	Q9: Will radiotherapy cause hair loss?	8	6.38	10.63	.04	0.5
	Q10: Will radiotherapy cause permanent damage?	8	6.75	10.25	.12	0.4

Based on the analysis of Krippendorff α and Fleiss κ coefficients across the 3 dimensions (general information; planning and treatment; and side effects), the results indicated a low level of agreement in the classification of questions for both GPT-3.5 and GPT-4. This trend of weak agreement was consistent across the overall set of queries in Multimedia Appendix 3.

### Consistency and Similarity

Regarding similarity and consistency, a cosine similarity score ranging from 0 to 1 was calculated, as previously described. Concerning similarity, the median (IQR) cosine similarity between GPT-3.5 and GPT-4 responses was 0.81 (IQR 0.05), indicating a reasonably good similarity between the 2 versions of ChatGPT. Notably, question 11 in the planning and treatment dimension exhibited the lowest similarity, with a value of 0.68. With respect to consistency, the cosine similarity median (IQR) for GPT-3.5 and GPT-4 responses were 0.85 (IQR 0.04) and 0.83 (IQR 0.04), respectively. In both versions, consistency was demonstrated to be good or very good, with values ranging between 0.74 and 0.92.

### Readability

The word count, sentence count, FRES, and FKGL score for both versions are summarized in Table 2. A relevant disparity was observed in the median (IQR) word count between GPT-3.5 and GPT-4 (299.00, IQR 176.5 versus 344.50, IQR 74.75). Additionally, the sentence count was higher in GPT-4 compared to GPT-3.5 (20.00, IQR 10.5 versus 18.00, IQR 17).

**Table 2.** Word count, sentence count, Flesch Reading Ease Score, and Flesch-Kincaid grade level score of responses from GPT-3.5 and GPT-4.

Dimension and questions^a^		GPT-3.5				GPT-4			
		Word count	Sentence count	FRES^b^	FKGL^c^	Word count	Sentence count	FRES	FKGL
**General information**									
	Q1	332	22	35.31	12.08	378	25	32.36	12.50
	Q2	414	27	35.97	12.05	453	28	35.97	12.26
	Q3	304	18	24.11	14.09	340	17	25.80	14.63
	Q4	188	7	13.53	18	268	15	25.81	14.10
	Q5	246	18	28.58	12.67	305	21	21.78	13.83
	Q6	378	27	35.96	11.72	431	27	36.55	12.13
	Q7	422	27	35	12.26	358	27	41.90	10.71
	Q8	389	22	32.74	13.09	351	16	30.79	14.41
	Q9	332	25	41.23	10.81	311	25	57.92	8.27
	Q10	84	5	17.56	14.98	223	16	21.59	13.71
	Q11	304	26	36.90	11.02	352	21	37.45	12.2
	Q12	178	7	33.21	14.94	298	15	47.28	11.60
	Q13	177	8	27.61	14.91	231	9	23.30	16.39
	Q14	348	22	35.92	12.18	410	13	19.24	18
**Planning and treatment**									
	Q1	374	28	49.19	9.72	402	30	52.44	9.27
	Q2	229	8	21.88	17.32	369	22	52.94	10.04
	Q3	165	8	15.25	16.99	359	20	16.19	10.93
	Q4	361	21	26.51	13.83	433	25	39.01	12.12
	Q5	316	16	16.79	15.82	378	24	26.80	13.43
	Q6	214	12	15.19	15.57	332	21	30.51	12.93
	Q7	358	22	34.35	12.51	472	27	48.93	10.78
	Q8	140	5	20.70	17.33	232	15	33.24	12.47
	Q9	361	27	40.94	10.87	416	31	43.54	10.52
	Q10	76	4	30.59	13.71	106	5	31.28	14.16
	Q11	164	6	0	18	314	16	33.07	13.52
	Q12	335	24	37.10	11.55	388	28	39.71	11.16
	Q13	247	16	37.38	11.88	315	19	37.73	12.12
	Q14	144	5	0	18	264	13	28.24	14.37
	Q15	327	24	52.01	9.39	337	22	62.50	8.35
	Q16	183	7	19.42	17.05	339	20	37.90	12.18
**Side effects**									
	Q1	340	26	48	9.81	324	11	26.28	16.91
	Q2	300	26	57.23	8.14	354	33	52.80	8.56
	Q3	150	7	36.19	13.54	270	9	32.57	16.17
	Q4	411	23	43.17	11.68	361	28	48.22	9.74
	Q5	397	21	17.81	15.47	418	17	13.49	17.49
	Q6	137	5	32.05	15.60	349	20	37.38	12.38
	Q7	298	21	45.09	10.50	371	27	38.28	11.33
	Q8	164	8	23.53	15.07	277	10	17.15	17.75
	Q9	108	5	43.91	12.50	214	15	57.94	8.72
	Q10	212	10	29.29	14.44	330	12	26.13	16.45

a Please refer to Table 1 for the full questions.

b FRES: Flesch Reading Ease Score.

c FKGL: Flesch-Kincaid Grade Level.

The FRES median (IQR) for GPT-3.5 and GPT-4 responses were 32.98 (15.59) and 34.61 (16.07), respectively. This indicates that the responses generated by the two versions were considered college-level and difficult to read. The FKGL median (IQR) for GPT-3.5 and GPT-4 responses were 13.32 (3.79) and 12.32 (3.32), respectively. This suggests that at least 13 years of education (college-level) are required to understand the responses generated by GPT-3.5, whereas the responses from GPT-4 require at least of 12 years of education (college-level) for comprehension.

## Discussion

### Principal Findings

The power and utility of AI platforms in health care, such as ChatGPT, are rapidly evolving and improving and have the potential to significantly improve patient education [5,49]. This study sought to assess the quality and reliability of ChatGPT responses to common patient queries regarding radiotherapy with the aim of determining its potential as a reliable source of information for patients. We also aimed to compare the performances of GPT-3.5 and GPT-4 in generating responses to the same radiotherapy-related queries.

Although most responses were correct or close to correct, upon comparing the accuracy of responses between GPT-4 and GPT-3.5 in the 3 dimensions, it became evident that GPT-4 consistently offered improved elucidation of specific concepts relevant to radiotherapy treatment. In question 10 of the general information dimension, GPT-4 specifically delineated that patients are nonradioactive and may safely interact with others posttreatment (“You can safely be around others, including children and pregnant women, without any risk of exposing them to radiation”). However, this aspect was not as clearly articulated in GPT-3.5, which failed to mention that patients may come into contact with others after treatment. Additionally, within the side effects dimension, in questions 2 and 3, GPT-4 emphasized that the intended creams to use throughout radiotherapy treatment should only be those recommended by the health care provider (“Apply a fragrance-free moisturizer recommended by your healthcare provider”) and specified strategies to mitigate fatigue, a treatment-related side effect. However, this advice was not as detailed in the responses from GPT-3.5. Within the planning and treatment dimensions, GPT-3.5 demonstrated a propensity to diverge from directly addressing the queried issue in certain responses in contrast to GPT-4. In question 7, the response did not describe the first day of treatment but rather outlined the entire course of the patient’s radiotherapy. Question 11 failed to specify the distance between the equipment and the patient, a detail that was thoroughly addressed by GPT-4. In response to question 12, GPT-3.5 did not describe what occurs during treatment, instead reiterating the patient’s overall course. This indicates that GPT-3.5 exhibits reduced accuracy when responding to queries related to planning and treatment, as Valentini et al demonstrated [50].

However, in GPT-4’s response to the 13th planning and treatment question, specific information was inaccurately presented as it erroneously stated that radiotherapy induces direct pain (“Direct Pain from Treatment Site: Radiotherapy can cause localized pain at the site of treatment”). This error may have occurred because not all web-based sources are reliable, and because the model is trained on a diverse array of internet texts, it may incorporate biased or outdated information. Consequently, misinformation regarding cancer continues to pose a significant concern in online communication, which could result in responses or recommendations that do not consider the most current, evidence-based medical practices [21].

Moreover, there were a few occasions in both versions in which a lack of information was demonstrated. For instance, in question 7 of the side effects dimension, neither version mentioned that radiation therapists, who are team members that assist the patient daily throughout their treatment [31], could serve as advisers for patients experiencing severe side effects.

In summary, both GPT-3.5 and GPT-4 demonstrated the ability to address concepts related to radiotherapy. However, GPT-4 provided more targeted and detailed responses, thereby exhibiting superior performance compared to GPT-3.5, as corroborated by several studies [9,24,25,43,47]. The reduced number of scores of 1 and 2 assigned by radiotherapy experts to GPT-4 responses indicated a substantial improvement in response quality and reliability.

### Comparison With Prior Work

In most responses, ChatGPT used a typical structure characterized by a succinct introductory paragraph, followed by 5 or 6 bullet points delineating the responses, culminating in a short concluding paragraph. Additionally, in a fair number of responses generated by GPT-3.5 (n=25) and GPT-4 (n=28), a statement was included advising that the information provided should always be discussed with health care providers, consistent with prior studies [43,51,52]. This recommendation is significant because the use of ChatGPT in health care must be carefully monitored and should not be viewed as a substitute for human judgment. Its performance, safety, and associated risks require thorough evaluation by experts before integration into mainstream practice [53]. Moreover, it is essential that the model be trained on a substantial dataset validated by experts. This rigorous validation process could enhance the reliability and trustworthiness of ChatGPT responses, ultimately benefiting patient care [54].

The cosine similarity score indicated a reasonably substantial similarity and consistency, and while subtle changes in sentence structure were noted, most answers remained consistent, implying accuracy [3].

A key feature influencing consistency is the temperature parameter, a value ranging from 0 to 2, which adjusts the randomness of each subsequent word in the chat output. A value of 0 results in minimal variability, whereas values approaching 1 introduce greater randomness and creativity into the responses. Creativity is a powerful tool in communication, as it simplifies complex concepts, fosters critical thinking, and enhances the accessibility of intricate information, making it especially valuable for developing patient education materials. However, using ChatGPT with high creativity settings in clinical contexts may present challenges. By lowering the creativity level, we ensure that the summarized information remains faithful to the training data, thereby prioritizing accuracy and reliability over creative expression. Although this feature is not currently available for modification in ChatGPT, it may be included in future iterations of the tool’s web interface [55].

Therefore, ensuring high reliability in ChatGPT’s outputs is essential for users to trust its data-driven conclusions. Although advances in ChatGPT’s performance can be attributed to key developments in its underlying technology, it is crucial that patients approach the information provided by AI tools such as ChatGPT with caution [56]. This is especially important given that ChatGPT does not disclose the bibliography used to generate responses [7,21,22]. This issue was observed during our study, as the bibliography was not disclosed in either version, indicating ChatGPT’s inability to inform users of the contentious nature of certain information [7,10,21,22]. This lack of transparency is particularly significant, given the ethical concerns that arise regarding its application in patient care. Its implementation may lead to unintended or undesirable issues such as risks of bias and transparency, challenges related to interpretability, and generation of inaccurate content, all of which can have serious negative consequences for patient health [53].

Moreover, patient accessibility to AI technology varies significantly according to socioeconomic status, education, age, and geography. Individuals in higher socioeconomic groups or urban areas have better access to the necessary infrastructure, whereas those in lower socioeconomic conditions or rural regions face significant barriers. The effective use of AI also requires digital literacy and computational skills, making the understanding of technology crucial [57]. Although AI can revolutionize education and research, GPT-4 may widen the gap between the wealthy and poor [58]. Conversely, the free availability of GPT-3.5 helps reduce socioeconomic inequalities in cancer treatment by providing fast medical information to all, regardless of financial circumstances [21].

Concerning readability, all responses were considered more difficult to read than the sixth grade reading level recommended for patient consumption, a concern highlighted in prior studies [4,37]. This finding suggests that although the content was predominantly accurate, it was presented at a level too advanced for the public, particularly for individuals with lower health literacy [37,59]. Health literacy, defined as a patient’s ability to read and understand health care information and make effective decisions, is crucial for quality patient engagement in health care options. An important aspect of health literacy is readability, which measures the ease with which text can be read and understood and is particularly relevant in radiotherapy due to the complexity of the field and its evolving nature. Patients with lower health literacy may have limited knowledge of radiotherapy; struggle to understand their conditions, treatments, and potential side effects; and often confuse different treatment modalities [60]. Owing to the heightened challenges faced by these patients, this bears particular significance [36].

Various studies have been conducted to assess ChatGPT’s ability to enhance readability and simplify responses [36,61-63], considering that, when providing patient education in a written form, it is important to ensure that it is tailored to the reading level of the target population [62]. To address this issue, it was suggested that direct prompts such as “Explain this to me like I am in fifth grade” could assist in generating simplified responses [36,61-63]. This indicates the potential of ChatGPT to tailor responses to varying literacy levels and customize them based on an individual’s educational background [61].

### Strengths and Limitations

This investigation revealed that both GPT-3.5 and GPT-4 demonstrated proficiency in addressing radiotherapy-related concepts, with GPT-4 exhibiting notably higher performance. Although GPT-4 achieved marginally better readability scores, the content generated by both models remains complex for a general audience. Therefore, their use should be complemented by strategies to improve their accessibility and readability. Moreover, ChatGPT holds significant potential in promoting health behavior changes among patients with cancer by enhancing health literacy and supporting the self-management of radiotherapy-related conditions [64]. However, ChatGPT’s responses must be validated by experts before they are integrated into a health care system to serve as a reliable source of information [53,54]. Therefore, ChatGPT shows promise in providing clinical guidance, suggesting treatment options, and serving as a valuable resource for medical education, facilitating a more effective shared decision-making process [6,47,59,65]. Hence, it can potentially serve as an alternative to current web-based resources [36].

This study had some limitations. First, the formulation and phrasing of queries in both versions may have influenced the performance of ChatGPT. Second, the queries were exclusively written in English, which restricted the responses to a single language. Third, although the total number of questions was comparable to other studies [1,8,11,18,47,50], the optimal number of queries needed to effectively evaluate the model remains undetermined, and the sample may not capture the full diversity of patient concerns about radiotherapy. Fourth, the scoring process inherently involves subjectivity, particularly with the GQS, as different raters may interpret and prioritize quality aspects differently. Fifth, the potential bias introduced by using a 5-point Likert scale may lead to a central tendency bias, as respondents tend to avoid extreme options and cluster their answers around the midpoint, which can limit the granularity of the evaluations and distort the data [66,67]. Sixth, this study was conducted within a specific time frame (April 2024), and ChatGPT is expected to improve continuously over time. Repeating this study at a later time could improve response quality.

Another limitation of our study was the limitations of ChatGPT itself. First, it should be noted that the information provided by GPT-3.5 is available only up to September 2021. Second, GPT-4 has a limited number of questions that can be posed within a specific time frame and it is exclusively accessible through paid subscriptions, potentially constraining the public’s access to more accurate information. Finally, ChatGPT is one of the many AI models available, making it uncertain whether the responses obtained represent the general characteristics of all LLMs.

### Future Directions

Further research is essential to fully comprehend ChatGPT’s role in patient education, including comparative studies with other AI models or traditional information sources, to better contextualize the findings. Additionally, future work should incorporate patient feedback into their understanding and satisfaction, providing valuable insights into the effectiveness of ChatGPT as an educational tool in real-world settings.

### Conclusions

Both GPT-3.5 and GPT-4 demonstrated the ability to address concepts related to radiotherapy, with GPT-4 exhibiting superior performance. Although GPT-4 achieved slightly better readability scores, the content produced by both versions remains challenging for the general public. This highlights the need for caution regarding potential misinformation and readability. Furthermore, the paid subscription model for GPT-4 could exacerbate existing health care disparities by limiting access to certain patient populations. Despite these limitations, ChatGPT shows promise as a valuable tool for addressing common patient queries regarding radiotherapy. However, its use should be complemented by strategies to improve accessibility and readability.

## Supplementary material

10.2196/63677 Multimedia Appendix 1 Flesch Reading Ease Score.

10.2196/63677 Multimedia Appendix 2 Flesch-Kincaid Grade Level score.

10.2196/63677 Multimedia Appendix 3 Analysis of Krippendorff α and Fleiss κ coefficients across the 3 dimensions.

## Abbreviations

AI: artificial intelligence
FKGL: Flesch-Kincaid Grade Level
FRES: Flesch Reading Ease Score
GQS: General Quality Score
LLM: large language model
