# Supplementary material for: Assessing the Quality and Reliability of ChatGPT’s Responses to Radiotherapy-Related Patient Queries: Comparative Study With GPT-3.5 and GPT-4
Source: JMIR Cancer. 2025 Apr 16;11:e63677. doi: 10.2196/63677 (PMC12017613; doi:10.2196/63677)
Supplement: Multimedia Appendix 1 [file cancer-v11-e63677-s001.docx]

### **Multimedia Appendix 1**

Flesch Reading Ease Score.

| **Score** | **Grade Level** | **Summary** |
| --- | --- | --- |
| 90 - 100 | 5th grade | Very easy to read |
| 80 - 90 | 6th grade | Easy to read |
| 70 - 80 | 7th grade | Fairly easy to read |
| 60 - 70 | 8th & 9th grade | Plain English |
| 50 - 60 | 10th to 12th grade | Fairly difficult to read |
| 30 - 50 | College | Difficult to read |
| 10 - 30 | College graduate | Very difficult to read |
| 0 - 10 | Professional | Extremely difficult to read |
