# Supplementary material for: Assessing the Quality and Reliability of ChatGPT’s Responses to Radiotherapy-Related Patient Queries: Comparative Study With GPT-3.5 and GPT-4
Source: JMIR Cancer. 2025 Apr 16;11:e63677. doi: 10.2196/63677 (PMC12017613; doi:10.2196/63677)
Supplement: Multimedia Appendix 2 [file cancer-v11-e63677-s002.docx]

### **Multimedia Appendix 2**

Flesch-Kincaid Grade Level Score.

| **Score** | **School Level** |
| --- | --- |
| 0 - 3 | Kindergarten/Elementary |
| 3 - 6 | Elementary |
| 6 - 9 | Middle School |
| 9 - 12 | High School |
| 12 - 15 | College |
| 15 - 18 | Post-Graduate |
