# Supplementary material for: Assessing the Quality and Reliability of ChatGPT’s Responses to Radiotherapy-Related Patient Queries: Comparative Study With GPT-3.5 and GPT-4
Source: JMIR Cancer. 2025 Apr 16;11:e63677. doi: 10.2196/63677 (PMC12017613; doi:10.2196/63677)
Supplement: Multimedia Appendix 3 [file cancer-v11-e63677-s003.docx]

### **Multimedia Appendix 3**

Analysis of Krippendorff's Alpha and Fleiss' Kappa coefficients across the three dimensions.

|  | | | Dimension | | | | | | | Global | |
| --- | --- | --- | --- | --- | --- | --- | --- | --- | --- | --- | --- |
|  |  |  | General Information | | Planning and Treatment | | Side Effects | | |  |  |
|  |  |  | ChatGPT 3.5 | ChatGPT 4 | ChatGPT 3.5 | ChatGPT 4 | ChatGPT 3.5 | ChatGPT 4 | ChatGPT 3.5 | | ChatGPT 4 |
| Krippendorff's Alpha Reliability Estimate | Alpha | | 0.02 | 0.2 | 0.21 | 0.18 | 0.02 | 0.13 | 0.11 | | 0.19 |
|  | 95% Confidence Interval | Lower bond | -0.09 | 0.10 | 0.11 | 0.07 | -0.11 | 0.00 | 0.04 | | 0.13 |
|  |  | Upper bound | 0.13 | 0.29 | 0.30 | 0.28 | 0.15 | 0.25 | 0.17 | | 0.25 |
| Feiss Kappa | Kappa | | -0.01 | 0.04 | 0.06 | 0.11 | -0.02 | 0.038 | 0.03 | | 0.09 |
|  | 95% Confidence Interval | Lower bond | -0.07 | -0.03 | 0.01 | 0.04 | -0.09 | -0.04 | 0.00 | | 0.05 |
|  |  | Upper bound | 0.06 | 0.11 | 0.11 | 0.18 | 0.05 | 0.12 | 0.07 | | 0.13 |
